# Complex Modulation of the *Aedes aegypti* Transcriptome in Response to Dengue Virus Infection

**DOI:** 10.1371/journal.pone.0050512

**Published:** 2012-11-27

**Authors:** Mariangela Bonizzoni, W. Augustine Dunn, Corey L. Campbell, Ken E. Olson, Osvaldo Marinotti, Anthony A. James

**Affiliations:** 1 Program in Public Health, University of California Irvine, Irvine, California, United States of America; 2 Department of Molecular Biology and Biochemistry, University of California Irvine, Irvine, California, United States of America; 3 Institute for Genomics and Bioinformatics, University of California Irvine, Irvine, California, United States of America; 4 Department of Microbiology, Immunology and Pathology, Colorado State University, Fort Collins, Colorado, United States of America; 5 Department of Microbiology and Molecular Genetics, University of California Irvine, Irvine, California, United States of America; Centro de Pesquisas René Rachou, Brazil

## Abstract

Dengue fever is the most important arboviral disease world-wide, with *Aedes aegypti* being the major vector. Interactions between the mosquito host and dengue viruses (DENV) are complex and vector competence varies among geographically-distinct *Ae. aegypti* populations. Additionally, dengue is caused by four antigenically-distinct viral serotypes (DENV1–4), each with multiple genotypes. Each virus genotype interacts differently with vertebrate and invertebrate hosts. Analyses of alterations in mosquito transcriptional profiles during DENV infection are expected to provide the basis for identifying networks of genes involved in responses to viruses and contribute to the molecular-genetic understanding of vector competence. In addition, this knowledge is anticipated to support the development of novel disease-control strategies. RNA-seq technology was used to assess genome-wide changes in transcript abundance at 1, 4 and 14 days following DENV2 infection in carcasses, midguts and salivary glands of the *Ae. aegypti* Chetumal strain. DENV2 affected the expression of 397 *Ae. aegypti* genes, most of which were down-regulated by viral infection. Differential accumulation of transcripts was mainly tissue- and time-specific. Comparisons of our data with other published reports reveal conservation of functional classes, but limited concordance of specific mosquito genes responsive to DENV2 infection. These results indicate the necessity of additional studies of mosquito-DENV interactions, specifically those focused on recently-derived mosquito strains with multiple dengue virus serotypes and genotypes.

## Introduction

The World Health Organization lists dengue as the most important arthropod-borne viral disease of humans [1]. The major vector of all four dengue virus serotypes (DENV1–4) is the cosmopolitan mosquito, *Aedes aegypti*. Close association with human populations and increasing intercontinental travel favor the expansion of its geographic distribution. There are no effective prophylactic and therapeutic drugs specific for dengue, and vaccine development is hindered by potential antibody-dependent enhancement that could put people at greater risk of life-threatening, severe dengue [2]. As a consequence, vector control is currently the only practical and effective strategy for disease prevention. The *Ae. aegypti* genome was sequenced and knowledge of genome-wide changes in patterns of gene expression following DENV infection is expected to identify genes involved in vector competence, the intrinsic ability of the mosquito to host and transmit DENV [3], [4]. This knowledge, coupled with germline transformation technology and anti-viral effector molecules, can be applied to the development of genetically-modified mosquitoes incapable of arbovirus transmission [5]–[7].

Although vertical transmission of dengue viruses has been reported, mosquitoes become infected mainly following ingestion of an infectious-blood meal [8], [9]. Viruses are transmitted to new human hosts during a subsequent bloodmeal following an extrinsic incubation period (EIP) of 7–14 days. The duration of the EIP depends on the mosquito strain, virus genotype and environmental factors [10]–[14]. During the first 1–2 days post infection (dpi), DENVs invade midgut epithelial cells through receptor-mediated endocytosis and initiate replication [15]–[18]. These processes involve both viral and host cellular factors [19]. Infection spreads laterally in the midgut epithelium to cells adjacent to those infected originally [13]. Virus titers peak in the midgut usually between 7–10 dpi and are followed by a decline [13], [20]. DENV infection disseminates from the midgut throughout the body, presumably through the tracheal system, reaching the salivary glands as early as 3 dpi [13]. Maximum virus titers in the salivary glands are reached 12–18 dpi. The saliva of an infected mosquito containing DENVs is injected in a human host during feeding to complete the transmission cycle.

*Aedes aegypti* populations of different geographic origin vary in their vector competence. Phenotypes include those in which DENVs either cannot establish a midgut infection (Midgut Infection Barrier) or cannot disseminate to other tissues (Midgut Escape Barrier) [11], [21], [22]. Other phenotypes manifest in the absence of virions in the saliva (Transmission Barrier). Differences in intensity of infection (peak titers) and duration of EIP also are observed among mosquito strains [13].

Multiple quantitative trait loci are associated with MIB and MEB, but specific genes have yet to be identified [11]. Mosquito genes and physiological pathways related to innate immunity, redox activity, energy production and metabolism are modulated in response to DENV infection [20], [23]–[29]. These observations come from multiple studies of specific mosquito tissues and time points following infection, and used different combinations of DENV2 genotypes and *Ae. aegypti* strains.

We investigated genome-wide changes in transcript accumulation in mosquito midguts, carcasses and salivary glands at 1, 4 and 14 dpi during the course of DENV2 infection. This analysis was conducted by RNA-seq with the Chetumal (CTM) strain of *Ae. aegypti* and DENV2-Jam1409. CTM was colonized recently (2005) from mosquitoes from the Yucatan Peninsula and is well-characterized for its response to non-infectious blood meals and for the kinetics of DENV2-Jam1409 infection [13], [30], [31]. A total of 397 genes had transcripts that showed statistically-significant differential accumulation following DENV infection, comprising both those found previously and those that are novel to this study, emphasizing the complex interaction between *Ae. aegypti* and DENVs [20], [24]–[29].

## Methods

### Mosquitoes

The *Ae. aegypti* Chetumal (CTM) strain derives from mosquitoes collected in Chetumal (Yucatan Peninsula, Mexico) in 2005 [30]. Mosquitoes were maintained at 28°C, 70–80% relative humidity, with 12–12 h light-dark photoperiod at Colorado State University (CSU; Fort Collins, Colorado). Larvae were fed on finely-ground fish food (Tetramin, Tetra Werke, Germany). Adult males and females were kept together in cages with unlimited access to water and a sugar source (raisins) until blood feeding. Defibrinated sheep blood (Colorado Serum Company, Denver, CO) was used for artificial blood meals.

### Virus and Mosquito Oral Infection

A DENV-2 strain, Jam1409, of the American-Asian genotype isolated in Jamaica in 1983, was used in this study [32]. DENV-2 Jam1409 was cultured in *Ae. albopictus* C6/36 cells and high passage (>25 passages) viruses were used in oral challenges [13]. Specifically, 350 and 330 adult females were fed either a DENV2-infected meal diluted 1∶1 in sheep’s blood or uninfected C6/36 cell culture medium diluted 1∶1 in sheep’s blood, respectively. Blood meals had a viral titer of 7.9 log10 pfu/ml. ‘DENVI’ or ‘B’ designate mosquitoes fed either the infected or uninfected blood meal, respectively. After blood feeding, 20 DENVI mosquitoes were sacrificed and viral titers determined for each individual using a standard method [33]. Specifically, mosquito bodies were homogenized in 270 µl of Dulbecco’s Modified Eagle Medium (DMEM) and then centrifuged to eliminate large debris particles. The supernatant was further filtered and used in serial dilutions to infect monolayers of Vero cells. The lowest concentration infecting Vero cells was used to calculate the viral titer of DENVI mosquitoes. Prevalence of infection was 65% and the median viral titer in infected mosquitoes was 2.2 log10 pfu per mosquito. Samples of 20–30 mosquitoes were removed at 1, 4 and 14 days post infection (dpi), and midguts were dissected from the carcasses. Midguts, salivary glands and corresponding carcasses were collected at 14 dpi. The time points of 1, 4 and 14 dpi were chosen as representing an early time point post-infection when viral particles are confined within the midguts, a time point during the dissemination out of the midgut and a time point within the peak of viral titers in the salivary glands, respectively [13]. Midguts and carcasses were collected individually for DENVI and in pools of 10 for B mosquitoes. Salivary glands were collected in pools of 20. Oral challenges and tissue dissections were conducted in a BSL-3 facility at CSU. All dissected samples were placed in 50 µl of 1∶1 PBS:Trizol, frozen in dry ice and shipped to the University of California, Irvine, for RNA extraction.

### RNA-seq Library Preparation and Sequencing

Total RNA was extracted with TRIZOL (Invitrogen) from pools of 5–10 midguts, 20 salivary glands and corresponding carcasses. The quality of the RNA was checked on an Agilent 2100 Bioanalyser and RNA from a total of 20–40 mosquitoes was pooled in equal amounts for RNA-seq library preparation. Illumina single-end RNA-seq libraries were prepared and sequenced for 40 cycles by the Expression Analysis Core at the UC Davis Genome Center. The need for biological replicates was mitigated as done previously by pooling samples of a large number of mosquitoes for each library [34]. Libraries were run at a concentration of 4–5 pM. Evidence for a correlation in transcript accumulation levels as estimated by RNA-seq data and quantitative real-time polymerase chain reaction (qRT-PCR) was provided previously [31].

### Data Analyses

RNA-seq data are deposited at NCBI's Sequence Read Archive (SRA) under accession number SRA058076. Reads were mapped to the Liverpool reference transcriptome (gene build AaegL1. 2) with the splice aligner TopHat, imposing a fragment length of 20 [35]. The accumulation levels of poly-adenylated RNAs were assumed to reflect changes in transcriptional activity of the corresponding genes and quantified in terms of mean number of reads per gene by DESeq [36], and in terms of Fragment Per Kilobase of exon model per Million mapped fragment (FPKM) using Cufflinks [37]. FPKM accounts for gene/transcript length and allows more accurate comparisons of accumulation levels across genes/transcripts within a sample.

Statistical significance in differential accumulation of poly-adenylated RNAs between DENVI and B mosquitoes at each time point/tissue was assessed conservatively by two different programs: DESeq and Cuffdiff within Cufflinks [36], [37]. DESeq requires the raw number of reads as input. As a consequence, DESeq was run at the gene level to avoid counting multiple times the reads mapping to exons shared by different transcript isoforms. Cuffdiff output comprises significance in accumulation levels of poly-adenylated RNAs both at the gene and transcript isoform level. Significance in differential gene product representation may not correspond unequivocally to significance at the transcript level because many genes encode a number of transcript isoforms. As a consequence, comparison of results between DESeq and Cuffdiff were done at the gene level. DESeq was run with the “blind” method, the “fit-only” sharing mode and the “local” fit type. Cuffdiff was run requiring a minimum alignment count of 10 and the upper-quartile-norm option, which can improve robustness of differential accumulation calls for less abundant transcripts. Both DESeq and Cuffdiff assess significance in differential accumulation fitting data to a negative binomial distribution, a method shown to maintain control of type-I error and that represents a statistical improvement with respect to the likelihood ratio test we applied previously [31], [36]. As a consequence, differences are expected in transcript quantification levels and significance in differential accumulation between CTM-B and -DENVI mosquitoes of this study and CTM-sugar fed and CTM-B mosquitoes at 5 hours post blood-meal [31]. Gene description used the biomart function of the EnsemblMetazoa (http://metazoa.ensembl.org/index.html) and Immunodb (http://cegg.unige.ch/Insecta/immunodb) [38], [39].

The motif-based sequence analyses suite MEME [40] was used to investigate the 2000 base-pairs (bp) adjacent to the 5′-end of ATG start-codon (hereafter designated “promoters”) of selected genes. MEME was run imposing 10 motifs, ranging from 6–20 bp. Each identified motif was analyzed for potential transcription-factor binding sites based on the hypothesis that sequence similarity reflects functional similarity. STAMP, a web tool for analyzing DNA-binding motif similarity, was used to search the closest match for each motif in the STAMP-supported transcription-factor databases [41].

## Results

### Rna Sequencing and Mapping Summary

Illumina RNA-seq technology was applied to study the accumulation levels of poly-adenylated RNAs at 1, 4 and 14 dpi in the carcasses and corresponding midguts of CTM females fed either a non-infectious (B) or DENV2-infected (DENVI) blood meal. Accumulation levels also were assessed in the salivary glands at 14 dpi. Single-end RNA-seq libraries were constructed starting from pools of 20–40 mosquitoes. Each RNA-seq library generated between 14 and 45 million 40 bp reads (Table 1). Sequenced reads were mapped by TopHat [35] to the *Ae. aegypti* transcriptome. The accumulation levels of specific poly-adenylated RNAs were compared between B and DENVI samples at each time point/conditions using DESeq [36] and Cufflinks [37] (Table S1). Genes whose products were identified as significantly differentially accumulated by DESeq are contained mostly within the larger number designated similarly by Cufflinks (Table 1). The Cufflinks results show that transcript isoforms increase the number of genes whose products are determined to accumulate differentially and significantly (Table S2).

**Table 1:** Summary of RNA-seq results.

			1 dpi		4 dpi		14 dpi		
			carcasses	midguts	carcasses	midguts	carcasses	midguts	salivary glands
Mapping^a^	DENVI	No.Mosquitoes	30	25	40	40	40	40	40
		No. reads	45,440,807	15,516,385	42,994,979	19,370,451	45,741,611	21,238,385	19,120,751
	B	No.mosquitoes	30	30	30	30	20	20	20
		No. reads	44,100,971	14,875,045	45,026,868	18,529,469	42,659,325	21,336,666	20,258,036
Differential transcript accumulation^b^		DEseq	13	31	63	96	73	14	257
		Cufflinks	369 (886)	774 (1063)	209 (537)	563 (934)	325 (567)	498 (841)	519 (861)
		Common	10	30	57	94	69	13	190

a Provides the number of mosquitoes dissected for each sample after either an infectious (DENVI) or non-infectious (B) bloodmeal in specific tissues at discrete days post infection (dpi). All mapping was to the *Ae. aegypti* transcriptome (AaegL1.2 gene build).

b Lists the number of genes identified by DEseq [35], Cufflinks [36] and common to both with transcripts accumulating differentially in the samples. The numbers in parentheses are the total number of transcripts isoforms detected as significantly differentially accumulated by Cufflinks.

### Comparisons of DENVI and B Mosquitoes

A total of 397 unique genes have poly-adenylated RNAs identified by both DESseq and Cufflinks as accumulated differentially and significantly between B and DENVI mosquitoes and we focus in detail on those here (Table S3). A high proportion (>46% in B and >38% in DENVI) of these genes has products with low read coverage (FPKM ≤15) in midgut samples. Higher proportions (>64%) with moderate-to-high read coverage (FPKM≥15) are seen with carcass and salivary gland samples of B mosquitoes (Table 2). The majority of these genes had products that accumulated more in B than DENVI mosquitoes.

**Table 2:** Classification of genes based on transcript accumulation level (in FPKM) after either an infectious or non-infectious blood meal.

Day post infectious bloodmeal	Tissue	Meal type^a^	FPKM≤1 ‘very low’^b^	FPKM≤15 ‘low’^b^	FPKM≤100 ‘moderate’^b^	FPKM≤1000 ‘high’^b^	FPKM≤10,000 ‘veryhigh’^b^	FPKM>10,000 ‘extremely high’^b^
1 dpi	carcasses	B	**0** (3962)	**0** (6098)	**4** (5060)	**5** (681)	**1** (101)	**0** (0)
		DENVI	**0** (4188)	**4** (5751)	**4** (5302)	**2**(625)	**0** (105)	**0** (1)
	midguts	B	**11** (7548)	**11** (5768)	**7** (1869)	**1** (538)	**0** (157)	**0** (22)
		DENVI	**7** (7492)	**19** (5938)	**3** (1859)	**1** (501)	**0** (100)	**0** (12)
4 dpi	carcasses	B	**5** (3734)	**9** (5188)	**22** (6068)	**19** (805)	**2** (106)	**0** (1)
		DENVI	**8** (3925)	**31** (5285)	**12** (5817)	**6** (781)	**0** (95)	**0** (1)
	midguts	B	**13** (6710)	**38** (6247)	**28** (2304)	**14** (515)	**1** (119)	**0** (7)
		DENVI	**24** (6701)	**36** (6223)	**29** (2359)	**5** (489)	**0** (123)	**0** (7)
14 dpi	carcasses	B	**7** (3778)	**23** (5224)	**24** (6006)	**14** (786)	**1** (107)	**0** (1)
		DENVI	**9** (3996)	**26** (5028)	**22** (5976)	**9** (799)	**3** (102)	**0** (1)
	midguts	B	**0** (6648)	**6** (6298)	**5** (2376)	**2** (450)	**0** (123)	**0** (7)
		DENVI	**2** (7024)	**3** (6142)	**7** (2172)	**1** (430)	**0** (126)	**0** (8)
	Salivary glands	B	**21** (6904)	**33** (6620)	**88** (1909)	**35** (338)	**12** (125)	**1** (6)
		DENVI	**96** (6154)	**72** (7046)	**19** (2226)	**3** (341)	**0** (117)	**0** (18)

a Specifies whether provided an infectious (DENVI) or non-infectious (B) bloodmeal.

b Genes whose poly-adenylated RNAs were or were not detected as significantly differentially accumulated are in bold and in parenthesis, respectively.

### Comparisons of Transcript Accumulation Levels in Infected and Uninfected Samples at 1 dpi

**Figure 1:**
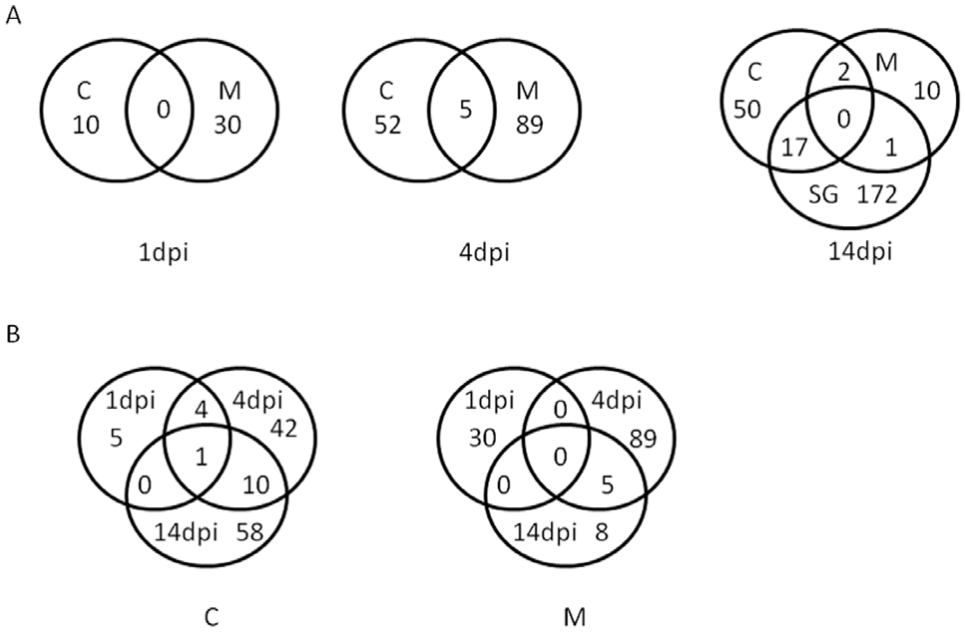
Numbers of genes whose transcripts accumulate differentially in response to dengue virus infection. (A) Venn diagrams with the number of genes whose transcripts accumulate differentially in response to dengue virus infection in carcasses (C), midguts (M) and salivary glands (SG) at 1, 4 and 14 days post infection (dpi). (B) Venn diagrams with the number of genes whose transcripts accumulate differentially in response to dengue virus infection in carcasses (C) and midguts (M) at different time-points.

**Figure 2:**
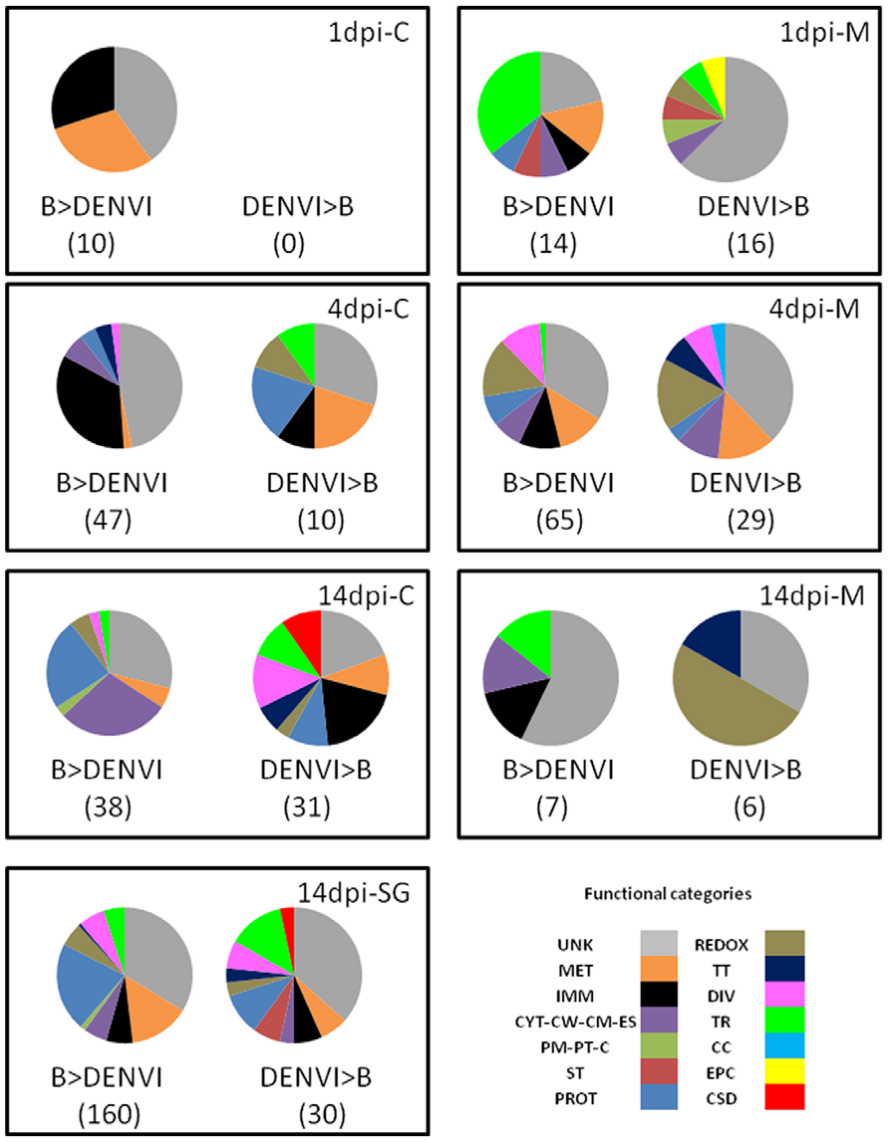
Functional categories of genes whose transcripts accumulate differentially in response to dengue virus infection in multiple tissues and times during infection. The functional categories for genes whose transcripts accumulate differentially in response to dengue virus infection are shown for each time point and tissue. The number of genes is shown in parentheses in each figure. Abbreviations for functional categories are: unknown (UNK), metabolism (MET), immunity (IMM), cytoskeleton, cell wall, cell motility and extracellular structures (C-CW-CM-ES), post-translational modification, protein turnover, chaperone (PM-PT-C), signal transduction (ST), proteolysis (PROT), oxidoreductase activity (REDOX), transcription and translation (TT), diverse (DIV), transport (TR), cell-cycle (CC), energy production and conversion (EPC), chromatin structure and dynamics (CSD). All other abbreviations are the same as Figure 1.

A total of 10 and 30 of the 397 genes had transcription products that accumulated differentially between B and DENVI in carcasses and midguts, respectively, at 1****dpi (Figure 1). Genes represented in the carcass samples generally had transcripts with higher read coverage than those in the midgut samples (Table 2). Transcripts of all of the genes represented in carcass samples were more abundant in B than DENVI mosquitoes (Figure 2), with the highest accumulation levels representing genes related to immunity (AAEL017536 [holotricin] FPKM_B_ = 1777.1; AAEL003841 [Defensin –A] FPKM_B_ = 220.6) and metabolism (AAEL001194 [fatty acid synthase] FPKM_B_ = 431.1, AAEL005790 [malic enzyme] FPKM_B_ = 363.2). Among the midgut samples, 14 genes had transcripts that were more abundant in B than DENVI mosquitoes, and 16 were higher in DENVI than B mosquitoes (Table S3). These genes are linked to 10 different functional categories (Figure 2). The gene encoding the antimicrobial peptide, Cecropin (AAEL017211), showed the highest transcript accumulation in B mosquitoes (FPKMB = 447.2). Genes with the highest transcript accumulation in midguts of DENVI mosquitoes were mainly of unknown functions or associated with oxidoreductase (redox) (AAEL009076 [NADH-ubiquinone oxidoreductase chain 4] FPKM_DENVI_ = 340.3) and transport (AAEL001503, FPKM_DENVI_ = 66.2) activities (Table S3).

### Comparisons of Transcript Accumulation Levels in Infected and Uninfected Samples at 4 dpi

Differential transcript accumulation at 4 dpi was seen for 146 of the 397 genes (Table S3). These included 52, 89 and 5 genes represented in carcass, midgut or overlapping samples of both, respectively. The latter five genes include two related to immunity (AAEL005641 [CTLGA5], AAEL006704 [FREP18]) and three (AAEL017380, AAEL003821, AAEL007703) of unknown function. All except AAEL017380 show higher accumulation in B than DENVI samples.

The majority (47 of 57) of genes represented in the carcass samples had transcripts that were more abundant in B than DENVI mosquitoes, and most were associated with immunity, a profile similar to that seen at 1 dpi (Figure 2). The immunity-related genes encode antimicrobial-peptides (AAEL0015515 [CECG], AAEL000627 [CECA], AAEL000598 [CECD], AAEL000611 [CECE], AAEL000625 [CECF], AAEL000621 [CECN], AAEL003832 [DEFC], AAEL003841 [DEFA], AAEL003857 [DEFD], AAEL003389 [ATT], AAEL004522 [Gambicin]), lysozyme (AAEL003723), a C-type lectin (CTLGA5, AAEL011610 [CTLGA7]) and fibrinogen-related proteins [FREP18]). The highest transcript accumulation in the carcasses was seen with DEFA (FPKM_B_ = 855) (Table S3).

Ten genes had products that accumulated more in carcasses of DENVI than B mosquitoes. The genes with the highest transcript accumulation levels were associated with redox (AAEL009018 [cytochrome P450] FPKM_DENVI_ = 308.9) and metabolism (AAEL007653 [allantoinase] FPKM_DENVI_ = 81.4, AAEL005752 [lysosomal alpha-mannosidase] FPKM_DENVI_ = 51.4) activities. Transcripts encoding the single isoform of the antimicrobial-peptide holotricin, which were detected as more abundant in B than DENVI mosquitoes at 1****dpi, showed higher levels of accumulation in DENVI mosquitoes at 4****dpi.

The majority (65 of 94) of genes represented in the midgut samples at 4 dpi had transcripts that were more abundant in B than DENVI mosquitoes, a pattern consistent with that seen with the carcass samples. Redox activities, metabolism and immunity were the most represented functional categories among these genes (Figure 2). Represented immunity-related genes did not encode antimicrobial peptides, but rather serine protease inhibitors (AAEL003182 [SRPN26], AAEL002704 [SRPN23]) and Clip domain serine protease (AAEL002124 [CLIPD6], AAEL001098). Similar to the 4 dpi carcass samples, FREP18 and CTLGA5 also were represented in the midguts. None of these genes had high accumulation levels (FPKM_B_ ≤22), apart from FREP18 (FPKM_B_ = 1815.9).

The highest transcript accumulation levels in midgut samples of B mosquitoes were associated with genes encoding redox proteins (AAEL014617, AAEL014609, AAEL007024, AAEL014607 [cytochrome P450] with FPKM_B_ of 425, 301.3, 172.4 and 164.3, respectively), those involved in metabolism (AAEL004027 [glucose dehydrogenase] FPKM_B_ = 282.7), linked to the cytoskeleton (AAEL001673 [actin] FPKM_B_ = 210.2) or diverse functions (AAEL017320, AAEL003123 with FPKM_B_ = 243.2 and 233.7, respectively).

The 29 genes whose products were more abundant in midguts of DENVI than B mosquitoes also were associated with metabolism, redox activity and the cytoskeleton, but not with immunity (Figure 2). Transcript accumulation levels of these genes were generally lower (FPKM_DENVI_ ≤81.7) than those of gene transcripts accumulated more in midguts of B versus DENVI mosquitoes (Table S3).

### Comparisons of Transcript Accumulation Levels in Infected and Uninfected Samples at 14 dpi

Differential transcript accumulation was seen for 252 genes at 14 dpi, the majority of which were in samples derived from salivary glands (Figure 1). The gene encoding the tubulin beta chain (AAEL002848) was the only one for which transcripts accumulated differentially in both midgut and salivary gland samples and it had a similar accumulation profile in the two tissues (Table S3). A total of 17 genes, associated mainly with proteolysis and transport activities, had differential transcript accumulation in both carcasses and salivary glands. Trends in expression profile were similar between the two tissues, with levels of accumulation tending to be higher in salivary glands (Figure S1).

**Figure 3:**
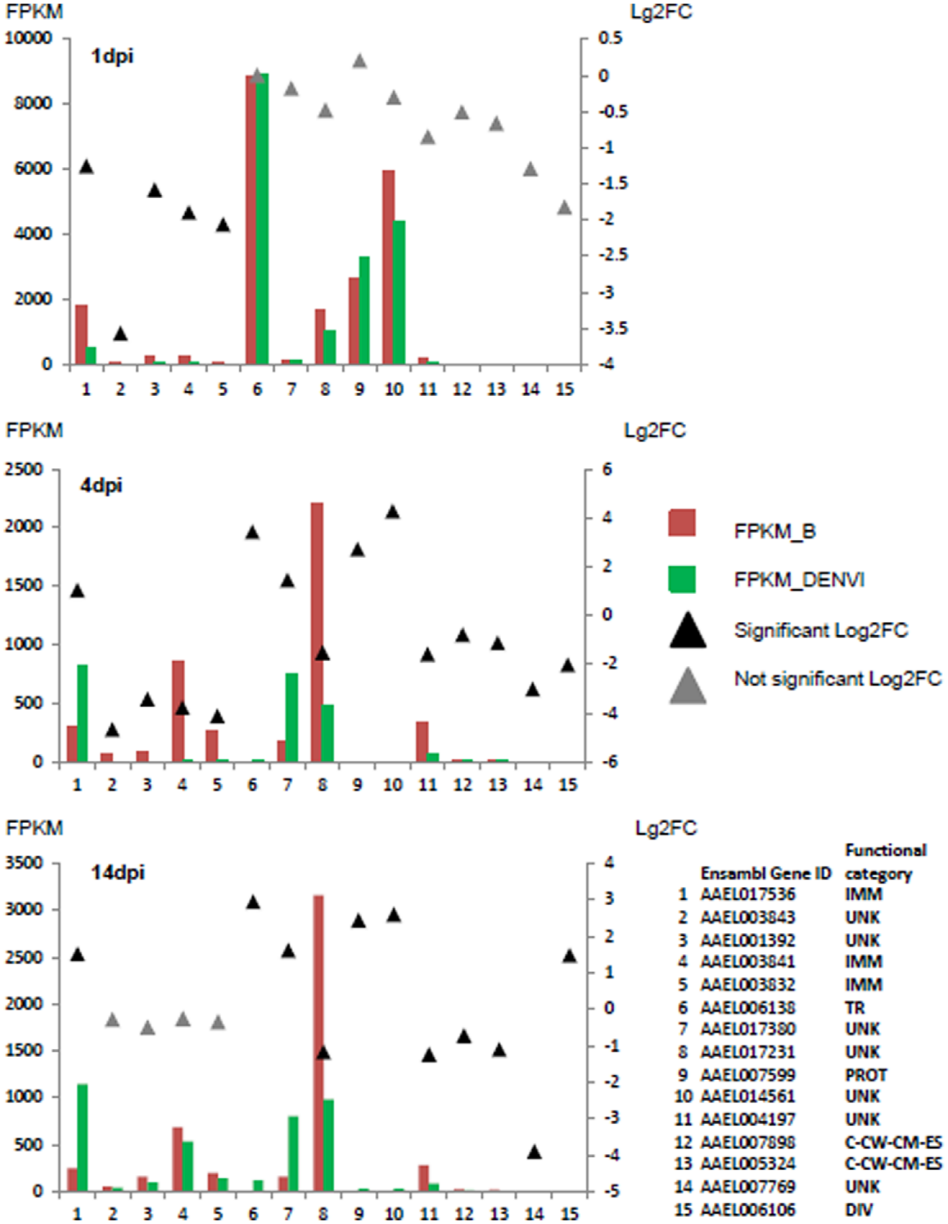
Genes whose transcripts accumulate differentially in carcasses throughout the course of dengue infection. FPKM values (colored bars) and Log2-fold changes in accumulation levels (filled triangles) are plotted on the left and right *y*-axes, respectively. Individual genes are listed by Ensembl Gene ID numbers and represented by the numerals on the *x*-axis. Abbreviations for the functional categories of each gene are the same as Figure 2.

The number of genes in carcass samples showing higher transcript abundance in B than DENVI mosquitoes (38) was similar to those more abundant in DENVI than B mosquitoes (31), and the differential accumulation tended to be higher for the latter, in contrast to what seen at 1 and 4 dpi (Table S3). Functional categories associated with the corresponding genes also differed from those seen at 1 and 4 dpi (Figure 3). Among the 38 genes whose products were more abundant in B than DENVI mosquitoes, the highest accumulation levels were observed in those related to the cytoskeleton (AAEL005961 [actin], FPKM_B_ = 314.6; AAEL002759 [tropomyosin] FPKM_B_ = 130.2) and proteolysis (AAEL007818 [trypsin 3A1 precursor], FRPKM_B_ = 239; AAEL003060 [serine-type endopeptidase], FPKM_B_ = 57.4). Among the 31 genes whose products were more abundant in DENVI than B mosquitoes, the highest accumulation levels were detected among those associated with chromatin structure and dynamics (AAEL003673 [histone H4], FPKM_DENVI_ = 1243.7; AAEL003689 [histone H4], FRPKM_DENVI_ = 1167.1, AAEL003669 [histone H2), FPKM_DENVI_ = 404), proteolysis (AAEL002610 [serine protease], FRPKM_DENVI_ = 401.9), transcription and translation (AAEL005004, FPKM_DENVI_ = 198.1) and immunity (AAEL011455 [CTLMA12], AAEL007599, AAEL007585, AAEL012216, AAEL015312 [cathepsin B], AAEL017536 [holotricin]) (Table S3).

The midgut samples showed 13 genes with differential transcript accumulation levels in B versus DENVI. Among the seven genes with higher transcript abundance in B samples than DENVI, the largest transcript accumulation levels were for genes of unknown function (AAEL004197, FPKM_B_ = 530.2; AAEL010675, FPKM_B_ = 123.8). The highest transcript accumulation levels among genes expressed more in DENVI than B were for those linked to transcription and translation (AAEL003603, FPKM_DENVI_ = 94.1), redox activity (AAEL007669, FPKM_DENVI_ = 53.1) or unknown functions (AAEL001702, FPKM_DENVI_ = 209.4; AAEL017571; FPKM_DENVI_ = 63.5). One immunity-related gene (AAEL003389 [ATT]) had transcripts representing its single isoform that were more abundant in B than DENVI mosquitoes of the midgut samples, a pattern similar to carcasses at 4 dpi.

Eighty-four percent (160/190) of genes modulated by DENV infection represented in the salivary gland samples had transcripts that were more abundant in B than DENVI mosquitoes. Their transcript accumulation levels tended to be higher than those observed for carcass and midgut samples (Table 2). This result is unlikely a bias of pooling RNA from salivary glands for RNA-seq library preparation because the corresponding dissected tissues from the same mosquitoes were used for the midgut and carcass library preparation. For example, 13 genes associated with proteolysis activities, metabolism or unknown functions had transcript accumulation levels of FPKM_B_ >1000 (Table S3). Fifty-five genes had read-coverage in salivary gland samples of B mosquitoes, but not in the salivary gland of DENVI mosquitoes, including three immunity-related genes (AAEL015136 [Niemann-Pick Type C-2, putative] FPKM_B_ = 35.4 and AAEL009531 [Niemann-Pick C1] FPKM_B_ = 15.5; AAEL01734 [Toll9B] FPKM_B_ = 30.4). Five other immunity-related genes had transcripts that were more abundant in salivary gland samples of B than DENVI mosquitoes (i.e. AAEL006699 [FREP34] FPKM_B_ = 64; AAEL006702 [FREP33] FPKM_B_ = 61.8; AAEL006704 [FREP18] FPKM_B_ = 778.4; AAEL005293 [GALE8A] FPKM_B_ = 247.4; AAEL013441 [Toll9A] FPKM_B_ = 22.3).

Two immunity-related genes were represented among the 30 gene products that were more abundant in salivary glands of DENVI than B mosquitoes (AAEL015312 and AAEL012216, both encoding for cathepsin B) (Table S3). A total of 12 genes had read coverage in salivary glands of DENVI mosquitoes, but not in the salivary gland of B mosquitoes and were associated with various functions in metabolism (AAEL008418 [pyrroline-5-carboxylate reductase] FPKM_DENVI_ = 21.4), proteolysis (AAEL013857, FPKM_DENVI_ = 14.3), the cytoskeleton (AAEL000335 [lamin] FPKM_DENVI_ = 14.2), redox activity (AAEL003211, FPKM_DENVI_ = 11.9), chromatin structure and dynamics (AAEL003950 [helicase] FPKM_DENVI_ = 10.1), transcription and translation (AAEL002422 [cytoplasmic polyadenylation element binding protein] FPKM_DENVI_ = 10.2) and signal transduction (AAEL015328, FPKM_DENVI_ = 12.5).

### Carcass-specific Differential Transcript Accumulation during the Progression of Dengue Infection

Limited overlap across time-points was detected in genes whose transcripts exhibited differential accumulation in carcass samples. AAEL017536, encoding holotricin, had significant differences in transcript abundance at all time points in carcass samples of B and DENVI mosquitoes, but showed opposite directions of differential accumulation at 1 and 4–14 dpi (Figure 3). Four genes encoding antimicrobial peptides DEFA and DEFC or associated with unknown functions (AAEL001392 and AAEL003843) had greater transcript abundance at 1 and 4 dpi in carcasses of B mosquitoes. The abundance of all four increased also in DENVI mosquitoes by 4 dpi and the statistical significance in differential accumulation was lost (Figure 3).

Ten genes exhibited differential transcript accumulation in carcass samples at both 4 and 14 dpi (Figure 1). Large differential accumulation profiles were evident from 1 to 4 dpi, but the profiles of most were similar between 4 and 14 dpi (Figure 3). For example, AAEL017380 increased 6-fold in abundance by 4 dpi in carcasses of DENVI mosquitoes while AAEL004197 increased 2-fold in B. The accumulation levels evident at 4 dpi then were maintained at 14 dpi. Transcript levels for genes AAEL006138, AAEL0014561 and AAEL007599 decreased by 4 dpi (10^5^–10^3^ fold in B and 10^3^-fold in DENVI) and remained low at 14 dpi. These three genes, despite their lower levels of accumulation at 4 and 14 dpi, were accumulated differentially between B and DENVI at the later stages of DENV infection. Read coverage for AAEL017231 increased in B 1.3-fold from 1 to 4 dpi and an additional 1.4-fold from 4 to 14 dpi; while coverage in DENVI decreased 2-fold from 1 to 4 dpi, but increased 2-fold from 4 to 14****dpi.

### Midgut-specific Differential Transcript Accumulation during the Progression of Dengue Infection

A total of 132 genes of the 397 had significant differential transcript accumulation levels in midgut samples between B and DENVI and only five of them maintained significant differential accumulations at 4 and 14 dpi (Figure 1.B). Two of these genes (AAEL001702 [unknown function] and AAEL001054 [GSTD4]) showed progressively higher transcript accumulation levels in DENVI samples from 1 to 14 dpi. The opposite trend was observed for AAE007776 [unknown function]. The accumulation of transcripts of the single isoform of gene AAEL007669 [redox] increased in abundance in midguts of DENVI mosquitoes from 1 to 4 dpi, but dropped at 14 dpi, and the AAEL014613 [cytochrome P450] transcript showed a peak in abundance in midguts of B mosquitoes at 4 dpi (Table S3).

### Discovery of *cis*-regulatory Elements

Genes with similar expression profiles may share common regulatory mechanisms, including the conservation of *cis*-regulatory elements (CREs) [42]. Evidence of this shared regulation may be evident in the control DNA sequences of mosquito genes that are expressed exclusively or highly-induced following dengue virus infection. Some of these genes also may have transcripts that accumulate to high or higher levels following an uninfected bloodmeal (B samples). The preferred expression profile for an antiviral effector gene would be one that is induced highly after blood feeding and is either further elevated or not affected by virus infection.

A total of 2012 genes showed read-coverage in salivary glands of DENVI mosquitoes but not in the salivary glands of B mosquitoes. While fifteen of these genes were associated with lipid metabolism, the majority were related to transcription and translation and/or chromatin structure and dynamics (Table S4). Forty-one of these had FPKM_DENVI_≥15, and only one, AAEL005034, showed tissue-specific expression (Table S1). As many as 762 and 1324 genes had transcripts that were detected in infected but not in the corresponding uninfected carcasses and midguts samples, respectively. The vast majority of these had FPKM_DENVI_<4. The exceptions were AAEL011066, AAEL010034 and AAEL002899, all encoding hypothetical proteins. At 1****dpi, AAEL011066 had read coverage only in carcass of DENVI (FPKM_DENVI_ = 14.97); AAEL010034 and AAEL002899 in midguts of DENVI (FPKM_DENVI_ = 8.11 and 6.56, respectively) (Table S1). The putative promoter regions of 41 genes with FPKM_DENVI_≥15 in salivary glands were analyzed using MEME [40], but no conserved CREs were identified (Figure S2).

We refined the search for conserved CREs by considering the promoters of genes whose products accumulated highly (FPKM_DENVI_≥100) in DENVI mosquitoes and were at the same or lower levels (FPKM_B_≤100) in B mosquitoes (Table S4). The latter criterion eliminates all genes whose accumulation levels are lowered in the presence of a dengue infection. In addition, this grouping also contains genes whose transcripts do not have a significant differential accumulation between infected and uninfected mosquitoes. The results support the presence of a module composed of six motifs (Figure S3: motifs 2, 4, 5, 6, 8, 10) in 4/51 genes with FPKM_DENVI_>100 in midguts at 1 and 4 dpi. The four genes sharing this module (AAEL007162 [APG8], AAEL001593 [glycerol-3-phosphate dehydrogenase (G3PDH)], AAEL003046 [saponin], AAEL000291 [V-type proton ATPase 16 kD proteolipid subunit (V-ATPase)]) had transcripts that were more abundant (1.03–1.64 fold) in DENVI than B mosquitoes in midguts samples at 1 and 4 dpi. These genes are not related functionally, but are associated with lipid metabolism, which has been shown to play an important role during DENV life cycle [19]. Furthermore, G3PDH links carbohydrate and lipid metabolism. The V-ATPase may be required to maintain transmembrane charge differential for virus entry. Saponin is a regulator of lipid degrading enzymes [43]. These genes, with the exception of that encoding G3PDH, had transcripts with FPKM_DENVI_>100 also in carcasses and salivary glands, but were not consistently higher or equally-abundant in DENVI than B mosquitoes. Transcripts encoding the V-ATPase were more abundant in DENVI mosquitoes of the susceptible MOYO-S strain three hours post infection (hpi) with DENV2 Jam1409 [27]. Matches to transcription factors in the TRASFAC- database [44] and supported by an e value ≤ e-05 were identified for motifs 4, 5, 6 and 10. The highest match of Motif 4 is Blimp1, which is an ecdysone-inducible transcription factor involved in *Drosophila* development, metamorphosis and oogenesis [45]. Motif 5 matched highest with Rel. The Rel or NF-κB superfamily of conserved eukaryotic proteins is involved in the control of immune and inflammatory responses, developmental processes, cellular growth and apoptosis. Motif 6 has high matches with High Mobility Group (HMG), Broad-Complex (BRC) and Forkhead transcription factors. HMG transcription factors are implicated in replication, recombination and DNA repair [46]. BRC is an ecdysone-regulated transcription factor implicated in developmental processes in *Drosophila* [47]. The Forkhead family of transcription factors includes seventeen sub-classes regulating development, homeostasis and reproduction in insects [48]. Motif 10 matches best with the NK-2-Nkx_TTF1 transcription factor, a homeodomain-containing transcription factor implicated in morphogenesis, differentiation and tissue-specific maintenance [49].

MEME analyses on the putative promoter regions of the 83 genes that had FPKM_DENVI_≥100 in carcasses consistently from 1 to 14 dpi showed the presence of three to five motifs (Figure S4: motifs 1, 2, 5, 7, 8) grouped tightly in eight genes (AAEL010097 [hypothetical protein], AAEL012629 [deoxyuridine 5′-triphosphate nucleotidohydrolase], AAEL008041 [bleomycin hydrolase], AAEL013068 [protein phsophatase-2a], AAEL017269 [novel protein coding], AAEL009604 [hypothetical protein], AAEL003552 [DNA-directed RNA polymerase subunit rpb6] and AAEL000739 [hypothetical protein]). Different combinations of these motifs are found in seven additional genes: (AAEL008849 [selenophosphate synthase], AAEL014903 [40S ribosomal protein S24], AAEL004484 [predicted protein], AAEL008768 [multiprotein bridging factor], AAEL009274 [hypothetical protein], AAEL001061 [glutathione transferase], AAEL012279 [eIF3j] and AAEL009320 [chaperonin]). This motif group is designated provisionally the “carcass module”. All 15 of these genes also had read coverage in midguts and salivary glands, but the abundance of the corresponding transcripts was not always as high (FPKM_DENVI = _≤100), and in some cases they are more abundant in B than DENVI mosquitoes. AAEL017269 and AAEL000739 had read coverage only in salivary gland samples of DENVI mosquitoes.

Motif 1 could be the binding site for the transcription factor MADS_MCM1+SFF_M01051 (e value of the match = 7.36e-06). The MADS-box family of transcription factors is conserved among yeasts, plants, insects, amphibians and mammals. It includes proteins associated with different biological roles (pheromone response, muscle-specific gene regulation, development) that operate generally by specifically recruiting other transcription factors into multi-component regulatory complexes [50]. Motif 2 has its best match to bZIP-type transcription factors (e value = 2.27e-06-). bZIP proteins belong to the largest and most conserved superfamily of transcription factors, the basic region leucine zipper transcription factors, involved in regulation of development, metabolism, and other cell functions such as secretion, oxidative stress and response to pathogens [51]–[53]. Motifs 5 and 7 match the *Arabidopsis thaliana* transcription factor trp_AtMYB-84_M00970 (e-value = 1.35–7.08e-10) and the Ras responsive element binding protein-1 (RREB-1) (e-value = 5.09e-9–1.38e-6), respectively, the latter of which regulates immunity and cancer-related gene expression in humans [54], [55].

**Figure 4:**
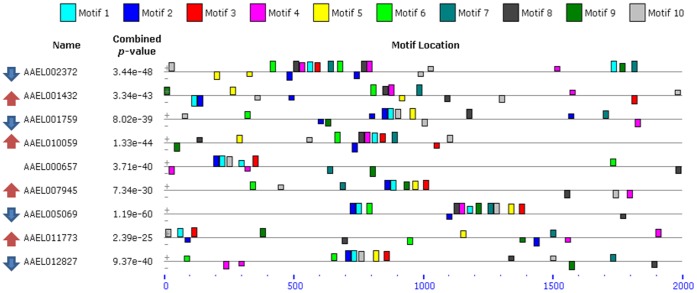
Examples of CRE discovery. MEME analysis of nine genes with FPKM_DENVI_≥100 in carcasses and salivary glands at 14 dpi. These genes also were identified with transcripts exhibiting significant differential accumulation in analyses of salivary gland samples of the Liverpool strain infected with DEV2 Thailand 16881 [26]. Colored boxes represent individual putative CREs and their locations in promoters of each gene. Red and blue arrows adjacent to Ensembl Gene ID indicate those genes whose transcripts were detected previously as more or less abundant following DENV infection [26]. Distances in base-pairs are provided below the schematic of each gene.

A total of 94 genes had FPKM ≥100 in DENVI mosquitoes in both carcass and salivary gland samples at 14 dpi, with corresponding values in B mosquitoes ≤100. The putative promoter regions of these genes analyzed by MEME revealed the presence of two groups of motifs (Figure S5: motifs 1, 2, 3, 4, 5, 7, 9, 10) co-occurring or alternating in 14 genes associated with diverse functions: AAEL001759 [40S ribosomal protein S9], AAEL005069 [ras-related protein Rab-1A], AAEL002372 [40S ribosomal protein S11], AAEL005165 [chaperone protein DNAj], AAEL000657 [hypothetical protein], AAEL005471 [Sec61 protein complex gamma subunit], AAEL001432 [protein disulfide isomerase], AAEL010059 [bacterial-type ABC transport ATP-binding subunit or RNAse l inhibitor], AAEL013407 [Catalase], AAEL007945 [eIF3 h], AAEL010169 [hypothetical protein], AAEL012827 [endoplasmin], AAEL011773 [calreticulin], AAEL010777 [TRX, putative]. These genes, except for AAEL002372, had high read coverage also in midgut samples, but not necessarily higher or equal accumulation in DENVI *versus* B mosquitoes. Some of these genes were detected previously as significantly differentially accumulated in the salivary glands of mosquitoes of the Liverpool strain (LVP) infected with DENV2 Thailand 16681 (Figure 4; [26]). Specifically, the transcription products of AAEL001432, AAEL010059, AAEL007945 and AAEL011773 were more abundant in salivary glands of infected LVP, while the opposite was observed for AAEL001759, AAEL005069, AAEL002372 and AAEL012827, which were more abundant in uninfected samples. AAEL00657 was listed as both up- and down-regulated following DENV infection, most likely at different time points, but this is not explicit in the report [26]. MADS_MCM1+SFF_M01051 and bZIP transcription factors were the best matches to motifs 1, 2, 5 and 7 (e value≤ e-5). Motif 3 has sequence similarity with motifs 5 and 7 of the previously designated “carcass module” and matches to trp_AtMYB-84_M00970 (e value = 2.25e-10) and RREB-1 (e value = 2.16e-7). The best match of motif 9 is to the HMG transcription factor (1.87e-5).

## Discussion

The dengue-susceptible strain CTM is well-documented in its ability to disseminate quickly viruses to peripheral tissues when compared to long-established laboratory strains [13]. We describe here changes in transcript accumulation levels in CTM during the course of a Jam 1409 DENV2 infection. Our analyses include multiple post-infection time-points and three tissues important for the viral transmission cycle: midguts, salivary glands and carcasses, the latter of which includes the fat body. The RNA-seq technology used in this study interrogates the whole *Ae. aegypti* transcriptome and allows for absolute quantification of poly-adenylated RNA levels. In general, more depleted rather than enriched transcript accumulation was observed. Importantly, hundreds of genes had exclusive read-coverage only in DENVI mosquitoes, but their accumulation levels were generally low (FPKM_DENVI_ <4 in carcass and midgut samples and <15 in salivary gland samples).

The “DENV down-regulation” trend is consistent to that observed with the Rockefeller (ROCK) strain infected by DENV2 New Guinea in transcriptional responses examined in whole-body (1–2, 7 and 14 dpi) or midgut samples (10 dpi) and in salivary glands of the LVP strain infected with DENV2 Thailand 16681 [20], [26], [28], [29]. These results support the hypothesis that dengue infection has a negative impact on fitness. However, a larger number of genes had products that increased instead of decreased in abundance in carcass samples 10 dpi and in salivary glands at 14 dpi in ROCK mosquitoes infected with DENV2-New Guinea [20], [28]. This trend also was evident in *Ae. aegypti* Aag2 cultured cells infected by DENV2 New Guinea and in both susceptible (Moyo-S) and resistant (Moyo-R) strains infected with DENV2 Jam1409 [24], [27]. These results emphasize the complex relationship among *Ae. aegypti* and dengue viruses.

We identified 397 genes whose products accumulate differentially and significantly between DENVI and B mosquitoes. Limited overlap of these genes across time-points and tissues was observed and this is consistent with previous reports [20], [28], [29]. Tissue-specific transcript accumulation of DENV-responsive genes was evident both in the number and the functional categories to which they belong. The largest number of DENV-responsive genes in midgut samples was detected 4 dpi, while in carcasses it was at 14 dpi. Overall, salivary glands samples showed the strongest response to DENV with respect to the number of genes represented, transcript accumulation levels and variety of functional categories elicited. These results likely reflect the long time during the EIP that the glands are infected. DENVs can be detected in CTM in the distal region of lateral lobes as early as 3 dpi, with the percentage of infected cells and the amount of viral antigen increasing up to 21 dpi [13]. Twenty-one days post infection is longer than the average survival of *Ae. aegypti* in laboratory conditions and in the wild [56], [57], supporting the conclusion that once infected, these mosquitoes remain infectious to humans throughout their life.

A total of 190 and 147 genes in CTM (this study) and ROCK (14 dpi; DENV2 New Guinea; [28]), respectively, were identified with differential transcript accumulation in salivary glands samples and only 28 of these overlap between the two studies. Furthermore, the direction of differential accumulation was conserved only for two immunity genes, those encoding cathepsin B (AAEL015312 and AAEL012216), which both show greater transcript abundance in DENVI than B mosquitoes. All other genes represented in common were more abundant in infected ROCK when compared to uninfected controls, but significantly less abundant in CTM when making the same comparison. Cathepsin B cysteine proteases are active in the low pH of the lysosomes, are involved in apoptosis of immune cells and required for Toll-Like-Receptor signaling in mammals [58], [59]. Cathepsin B in arthropods also participates in developmental processes such as metamorphosis and embryonic development [60]). Apoptosis may be required for Sindbis virus replication and dissemination in *Ae. aegypti* and cytopathology consistent with apoptosis was seen in the salivary glands of *Culex quinquefasciatus* infected with West Nile Virus (WNV) [61], [62]. Data obtained from studies in *D. melanogaster* support the conclusion that manipulating the apoptotic pathway can affect negatively RNA interference (RNAi), the most characterized antiviral mechanism acting in mosquitoes [23], [63].

Comparisons of differential transcript accumulation profiles in salivary glands with the CTM (this study) and ROCK and LVP strain (infected with DENV2 Thailand 16681; [26], [28]) show only four genes whose transcript abundance consistently varies following infection: AAEL002295 [Leucine Rich Transmembrane protein], AAEL009244 [serine-type endopeptidase], AAEL000647 [hypothetical protein], AAEL002263 [hypothetical protein]. The Leucine Rich Transmembrane motif is found commonly in proteins involved in signal transduction, including mosquito Tolls, and protein-protein interaction [64]. The serine-type endopeptidase AAEL009244 was less abundant in salivary glands following DENV infection in both CTM and LVP mosquitoes, but was more abundant in infected ROCK [26], [28]. Serine-type endopeptidases are proteolytic enzymes involved in digestion and immunity and are classified based on substrate specificity that result from sequence variation in amino acid moieties at their catalytic sites [65], [66]. Silencing of the midgut trypsin Aa5G1 (AAEL013712) was accompanied by an increase in infection of DENV2 Jam1409 in the Rexville D-Puerto Rico (Rex-D) strain [67]. Aa5G1 transcripts in our study were less abundant in CTM midgut samples at all time points, but not at statistically-significant levels. CTM and Rex-D strains differ in their susceptibility to DENV2 Jam1409 with the progression of the infection being more rapid and the intensity of infection higher in CTM [13]. The abundance of the Aa5G1 transcripts may be causal or a marker of this differential susceptibility.

Bioinformatic analysis of the conceptual translation product of the serine-type endopeptidase encoded by AAEL009244 supports higher similarity to the chymotrypsin, AaJA15 (AAEL001703), than to the trypsin, Aa5G1 [66]. Its role in DENV infection is unknown, but experiments that compare its expression profile along with that of Aa5G1 in CTM, LVP, Rex-D and ROCK strains infected with of the same DENV2 genotype could determine if there is a correlation of changes in accumulation levels of AaJA15 and Aa5G1, the progression of infection and resulting EIP.

Salivary gland samples had 23 and 21 additional genes whose transcripts showed consistent differential accumulation at 14 dpi in pair-wise comparisons of LVP and either CTM or ROCK, respectively. However, the directions of change were conserved in only nine and one genes, respectively [26], [28]. The use of different DENV2 genotypes among the three studies complicates interpretations of the effects of mosquito strain, virus genotype or both on the contrasting results. Furthermore, in addition to differences based on geographic origins, selection during laboratory colonization, including loss of polymorphism, could affect vector competence [68], [69]. The ROCK strain, colonized originally from the Caribbean, has been maintained in the laboratory since the 1930’s [70] and showed less variation in susceptibility to different DENV genotypes than mosquitoes derived recently from the wild, supporting the interpretation of a loss in variation of vector competence traits [69]. Different rates in the progression of infection and dissemination of the virus or a different timing in mosquito host gene expression also could account for the differences observed. Finally, differences in the techniques used (microarray, Digital Gene Expression and RNA-seq) could influence the degree of overlap among the represented genes and transcript accumulation profiles of the various combinations of mosquito strains and DENV genotypes, but cannot explain the opposite trends (higher or lower) in transcript accumulation in those genes whose products were consistently detected as significantly differentially accumulated between DENVI and B mosquitoes in the different combinations of mosquito strains-DENV2 genotypes.

### Innate Immunity Genes

Characterized antiviral mechanisms in mosquitoes include the RNAi machinery and the innate immune Toll and Janus kinase signal transducer and activator of transcription (JAK-STAT) pathways [20], [23], [27], [71]. Tissue-specific differences in immune response to DENV infection were observed in our study. All transcripts representing immunity-related genes with differential accumulation in midgut samples were always more abundant in B than DENVI mosquitoes, supporting the conclusion that there is a suppression of the insect immune system following infection. This result may reflect the general ‘DENV down-regulation trend” observed. A similar pattern was seen in carcass samples at early time points post infection, but the opposite was observed at 14 dpi, reflecting a possible change in immune modulation during the course of the infection. The C-type lectins CTLMA12 (AAEL011455) and CTLGA5 (AAEL005641), the toll receptors Toll9A (AAEL0013441) and Toll9B (AAEL011734), FREP33, 34 and 18 (AAEL006702, AAEL006699 and AAEL006704), the Leucine Rich Transmembrane protein encoding gene AAEL002295, the Serine protease inhibitor encoding gene AAEL002704, three cathepsin b genes (AAEL007585, AAEL0012216 and AAEL015312), gene AAEL009531 and five antimicrobial peptides (DEFC, DEFA, DEFD, CECG and Gambicin) showed differential transcript accumulation profiles at 10 dpi in carcasses, midguts and salivary glands of the ROCK strain infected with DENV2 New Guinea, at 18 hpi in MOYO-R infected with DENV2 Jam1409 and in salivary gland samples of LVP infected with DENV2 Thailand 1668 [20], [26], [27]. However, as we observed, the directions of changes in accumulation were not always conserved. These findings confirm the involvement of the Toll and JAK-STAT pathways in modulating DENV infection across different *Ae. aegypti* strains. However, the different timing during which the mosquito transcriptional response was studied prevents further comparison.

### Genes not Associated Directly with Immune Functions

We observed that genes encoding histones H2A (AAEL003669) and H4 (AAEL003673, AAEL003689) and helicase (AAEL003950) had differential transcript accumulation profiles at 14 dpi in either carcass or salivary gland samples, and were more abundant in DENVI than B mosquitoes. Differential transcript accumulation of several genes associated with chromatin structure and dynamics have been reported previously. For example, the core histone H3-encoding gene (AAEL003685) was more abundant in ROCK at 1, 2 and 7 dpi following challenge with DENV2, Yellow Fever virus and WNV [29]. Genes encoding histone deacetylase (AAEL001069, AAEL002528), histone H3 methyltrasferase (AAEL006783) and histone-lysine n-methyltrafersase (AAEL004290) exhibited differential accumulation at 18 hpi in either the MOYO-S or MOYO-R strains [27]. Six additional genes (AAEL015674 [histone H2B], AAEL015678 [histone H2B], AAE003827 [histone H3], AAEL003833 [histone H3], AAEL003823 [histone H4] and AAEL000490 [histone H4]) showed differential accumulation at 3 hpi in MOYO-S infected with DENV2Jam1409 [27]. Genes AAEL000149 [SAP30_AEDEE] and AAEL006661 [histone acetyltransferase, putative] were significantly less abundant in LVP salivary gland samples infected with DENV2 Thailand 16681 [26]. Some 23 genes encoding histone H3 (AAEL011424, AAEL003685, AAEL003827, AAEL003833, AAEL003836, AAE003850, AAEL003852, AAEL003856), histone H2A (AAEL003818, AAEL003820, AAEL003826, AAEL003851, AAEL003862, AAEL007609, AAEL012499, AAEL015390) and histone H4 (AAEL003673, AAEL003823, AAEL3838, AAEL003846, AAEL003863 and AAEL003866) had differential accumulation profiles in carcasses of ROCK infected with DENV2 New Guinea 10 dpi [20]. Although different genes or paralogues were identified from the various samples varying in time post-infection, host strain, and virus genotype, these results support a link between chromatin structure and dynamics and DENV infection. A number of viruses, including DENV, Epstein-Barr, *Herpes simplex* type 1, influenza and HIV-1, regulate host gene expression by affecting host chromatin structure through direct post-translational modification of histones or by interacting with other cellular proteins involved with chromatin function [29], [72]–[75].

Five genes encoding cytoskeleton-related products associated previously with DENV infection had transcripts in midgut samples that were less abundant in DENVI than B at 4 dpi. The actin-encoding genes, AAEL002495 and AAEL004616, showed differential transcript accumulation at 10 dpi and 14 dpi, respectively, in carcass and salivary glands samples of ROCK infected with DENV2 New Guinea [20], [28]. AAEL005317 [titin] and AAEL006911 had differential transcript accumulation at 3 and 18 hpi, respectively, in MOYO-S [27]. Modifications following DENV2 infection of the expression profiles of actin and other proteins involved in the maintenance of cytoskeleton and cell-to-cell junctions occur in human endothelial cells and are proposed to affect endothelial permeability [76].

The functional groups of metabolism, redox processes, proteolysis and transport are represented in our study in the majority (165 out of 397) of genes with differential transcript accumulation post DENV infection, and this confirms previous results [20], [27], [29]. Transcript abundance of 48 of these genes was reported as affected consistently by infection [20], [26], [27]. Remarkably, agreement in the direction of changes was limited primarily to genes encoding redox activity (i.e. AAEL007024, AAEL014609, AAEL014616 and AAEL007669) or linked to proteolysis (AAEL008619, AAEL002610). Transcripts corresponding to these genes were consistently more abundant following DENV infection in CTM midguts and carcasses, LVP salivary glands and ROCK carcasses and salivary glands 4, 10 and 14 dpi [20], [26], [28]. AAEL002610, encoding a protease, has transcripts that are more abundant following infection in carcass samples of ROCK and CTM at 10 and 14 dpi, respectively [20]. Two genes, AAEL007097 and AAEL010099, encoding phosphatases, showed divergent trends in transcript accumulation following virus infection. AAEL010099 transcripts were less abundant in CTM and LVP salivary gland samples, while AAEL007097 transcripts were consistently more abundant in CTM salivary glands and ROCK carcasses at 14 and 10 dpi, respectively [20], [26]. At this point we have no explanation of why genes encoding the same functional products show contrasting transcript accumulation profiles.

### Cis-regulatory Elements

The preferred promoter for an anti-dengue effector gene would be one that is induced following a bloodmeal and is not affected by viral infection. Furthermore, we hypothesize that genes with this preferred expression profile may share common regulatory mechanisms based on conserved CREs. Analysis of the 5′-end of genes whose transcripts exhibited accumulation profiles exclusively in salivary gland following DENV infection did not reveal conservation of CREs. The regulation of these genes is likely not coordinated through common promoter elements. In contrast, putative CREs were identified for genes whose transcripts were represented and modulated highly in response to dengue infection in midguts (1–4 dpi), carcasses (all time points studied) and salivary glands and carcasses (14 dpi). Several of the identified motifs showed high-quality matches to known transcription factors. The CREs likely are not related specifically to dengue infection since the majority of the genes included in the analyses also had read coverage in B mosquitoes. This observation does not affect the potential utility of the CREs as components of synthetic promoters to direct expression of anti-dengue effector molecules. Indeed, driving effector gene expression with control DNA that responds to an uninfected bloodmeal and either is unaffected or is enhanced by an infected bloodmeal could elicit a protective antiviral response in the mosquitoes. Remarkably, 10 genes whose transcripts were modulated following DENV2 16881 infection in LVP salivary gland samples were among the 14 genes with FPKM_DEVI_≥100 in our carcass and salivary gland samples that have a common group of CRE motifs [26]. However, not all of these ten genes had transcripts that were consistently more abundant following infection, and one gene, (AAEL00657), had transcripts reported as more and less abundant [26]. These results and the findings that several motifs are putative binding sites for transcription factors acting through repressors and activators support a complex model of transcription modulation that requires further investigation.

### Conclusions

The study of genome-wide changes in transcript abundance in *Ae. aegypti* following dengue infection is anticipated to identify genes and control DNA elements involved in vector competence. This knowledge is expected to contribute to development of novel vector control strategies. The interactions between the mosquito host and the virus are complex at both the individual and population levels. Many mosquito tissues are affected by the virus during the course of the infection and *Ae. aegypti* strains can show unique responses of the transcriptome in response to a blood meal and susceptibility to DENV infection [11], [31]. Additionally, the variation in DENV serotypes and genotypes contributes to genetically-distinct differences in vector competence [12], [77]. While we identified modulations in transcript abundance following DENV infection that represent genes encoding similar functional categories, a limited number of specific genes were concordant across the mosquito strains and DENV2 genotypes following comparison of our data with previous studies [20], [26]–[29]. Furthermore, the direction of changes in transcript abundance in samples from infected and uninfected mosquitoes was not always conserved among the various studies, an observation that we consider independent of the methodology used to assess differential transcript accumulation. These results support the need for a comprehensive analysis of dengue infection focusing on recently-colonized laboratory strains or wild-caught mosquitoes to capture most of the genetic variability at the host level and different DENV serotypes/genotypes [69]. This analysis also should account for possible variation in the progression of the viral infection and timing of host gene expression.

## Supporting Information

Figure S1 **Genes whose transcripts accumulate differentially in both carcasses (A) and salivary glands (B) at 14 dpi.** FPKM values (colored bars) and Log2-fold changes in accumulation levels (filled triangles) are plotted on the left and right *y*-axes, respectively. Individual genes are listed by Ensembl Gene ID numbers and represented by the numerals on the *x*-axis. Abbreviations for the functional categories of each gene are the same as Figure 2.
(TIF)

Figure S2 **Putative CRE discovery with 41 genes with exclusive read coverage in salivary glands of DENVI mosquitoes with FPKM_DENVI_>15 at 14 dpi.** Colored boxes represent individual putative CREs and their locations in promoters of each gene. Distances in base-pairs are provided below the schematic of each gene.
(TIF)

Figure S3 **Putative** **CRE discovery with 51 genes in midgut samples with FPKM_DENVI_>100 at 1 and 4 dpi.** Colored boxes represent individual putative CREs and their locations in promoters of each gene. Distances in base-pairs are provided below the schematic of each gene.
(TIF)

Figure S4 **Putative CRE discovery with 83 genes in carcass samples with FPKM_DENVI_≥100 from 1–14 dpi.** Colored boxes represent individual putative CREs and their locations in the promoters of each gene. Distances in base-pairs are provided below the schematic of each gene.
(TIF)

Figure S5 **Representative putative CRE discovery with 94 genes in carcass and salivary gland samples of DENVI mosquitoes with FPKM_DENVI_≥100 in 14 dpi.** Colored boxes represent individual putative CREs and their locations in the 2promoters of each gene. Distances in base-pairs are provided below the schematic of each gene.
(TIF)

Table S1 **Gene accumulation levels in FPKM across time points (1, 4 and 14 dpi) and tissues (C = carcass, M = midgut, SG = Salivary Gland) in dengue-infected (DENVI) (D) and uninfected (B) mosquitoes.**
(XLSX)

Table S2 **Correspondence between genes and transcript isoforms detected as significantly differentially accumulated between B and DENVI mosquitoes by Cufflinks.** The number of genes and their corresponding transcript isoforms detected by Cufflinks as differentially accumulated are compared. The number of genes that could not be distinguished and the number of transcript isoforms are shown in parenthesis.
(XLSX)

Table S3 **List of genes detected as significantly differentially accumulated by DESeq and Cufflinks.** Genes encoding for polyadenylated RNAs found differentially accumulated in carcass, midgut and salivary gland samples at 1, 4 and 14****dpi are listed with their accumulation levels in B (FPKM_B_) and DENVI (FPKM_DENVI_) mosquitoes. Genes with transcripts consistently differentially accumulated in previous studies are marked.
(XLSX)

Table S4 **List of genes with transcripts with FPKM_DENVI_≥100 and accumulated at the same level or less in B mosquitoes.** For the salivary glands the genes with transcripts with read coverage in DENVI mosquitoes, but not in B mosquitoes is also given; their functional attributes is reported in sheet2. The promoters, consisting of 2000 bp upstream the ATG of these genes were used an input for MEME analyses.
(XLSX)
