# Supplementary figures and images for: Complex Modulation of the Aedes aegypti Transcriptome in Response to Dengue Virus Infection
Source: PLoS One. 2012 Nov 27;7(11):e50512. doi: 10.1371/journal.pone.0050512 (PMC3507784; doi:10.1371/journal.pone.0050512)

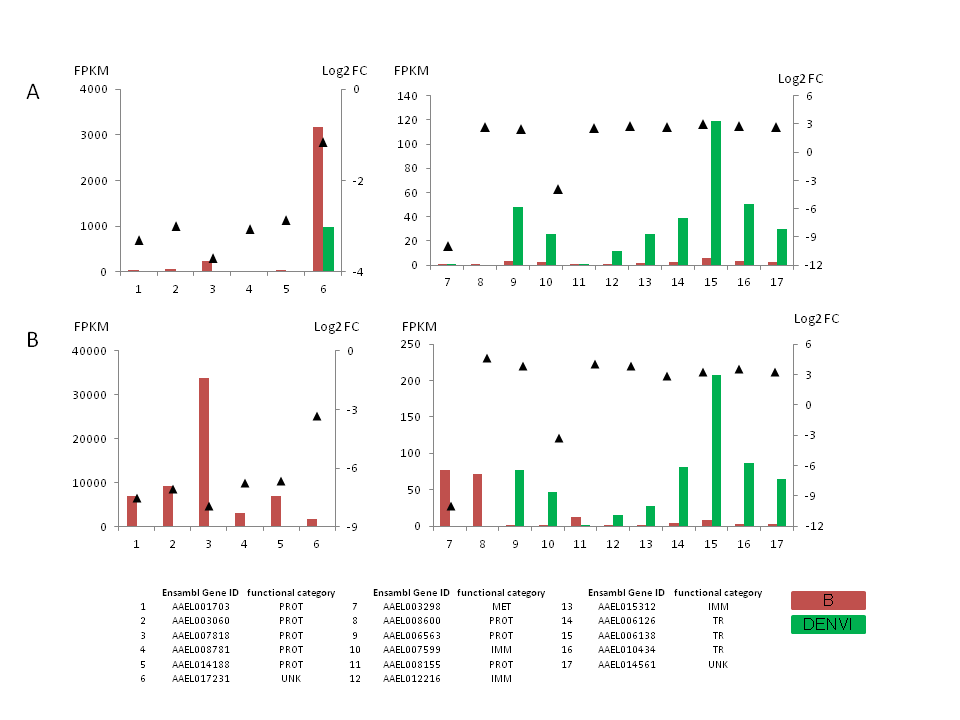

Supplement: Figure S1 — Genes whose transcripts accumulate differentially in both carcasses (A) and salivary glands (B) at 14 dpi. FPKM values (colored bars) and Log2-fold changes in accumulation levels (filled triangles) are plotted on the left and right y-axes, respectively. Individual genes are listed by Ensembl Gene ID numbers and represented by the numerals on the x-axis. Abbreviations for the functional categories of each gene are the same as Figure 2. (TIF) [file pone.0050512.s001.tif]

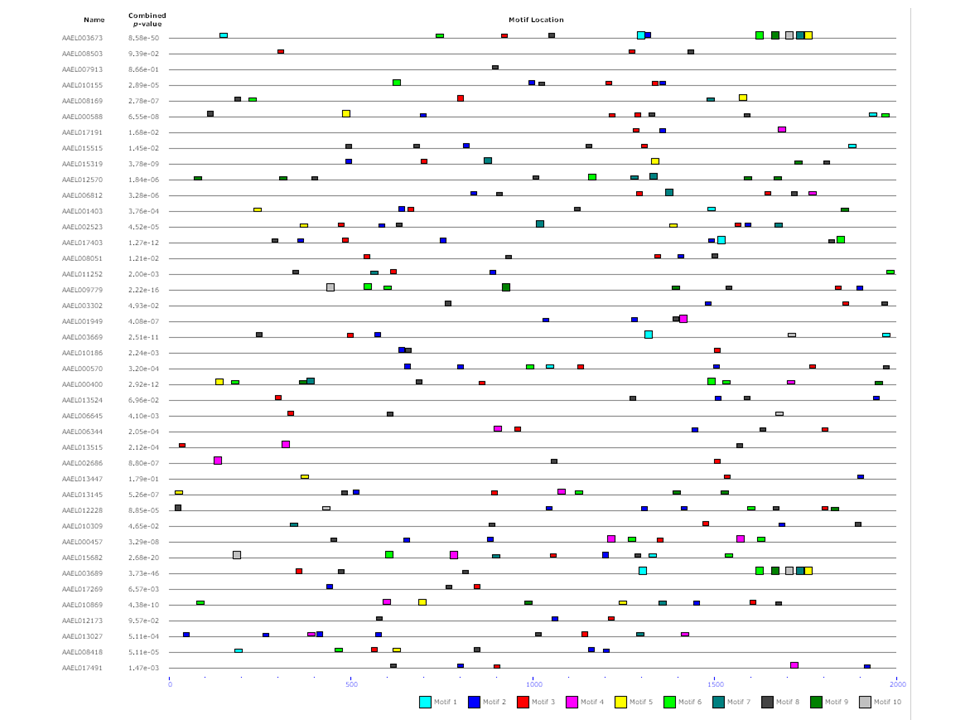

Supplement: Figure S2 — Putative CRE discovery with 41 genes with exclusive read coverage in salivary glands of DENVI mosquitoes with FPKMDENVI>15 at 14 dpi. Colored boxes represent individual putative CREs and their locations in promoters of each gene. Distances in base-pairs are provided below the schematic of each gene. (TIF) [file pone.0050512.s002.tif]

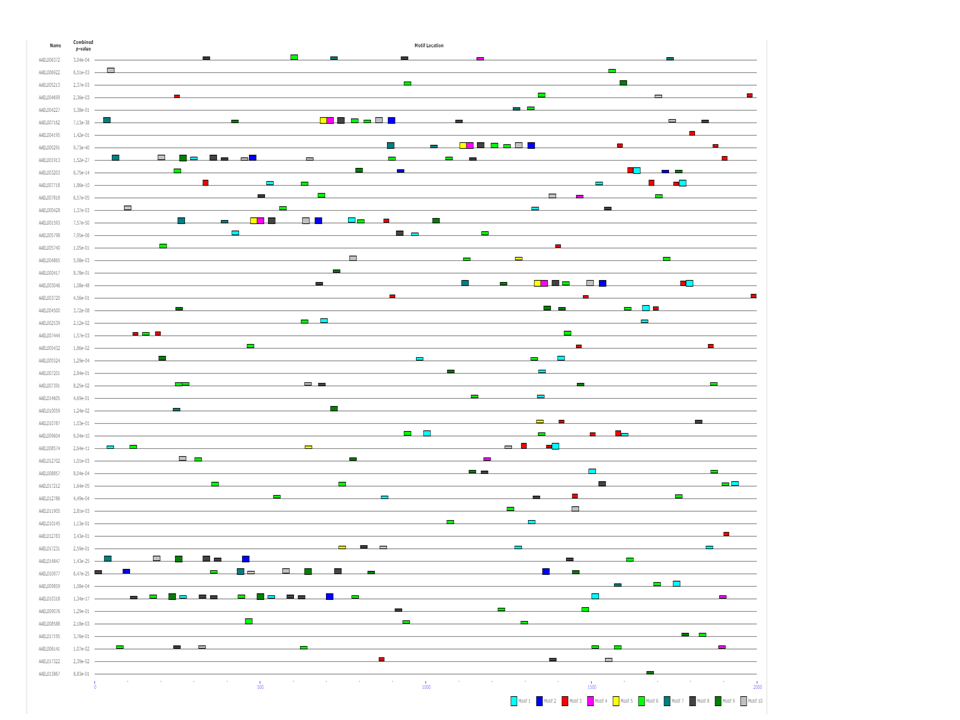

Supplement: Figure S3 — Putative CRE discovery with 51 genes in midgut samples with FPKMDENVI>100 at 1 and 4 dpi. Colored boxes represent individual putative CREs and their locations in promoters of each gene. Distances in base-pairs are provided below the schematic of each gene. (TIF) [file pone.0050512.s003.tif]

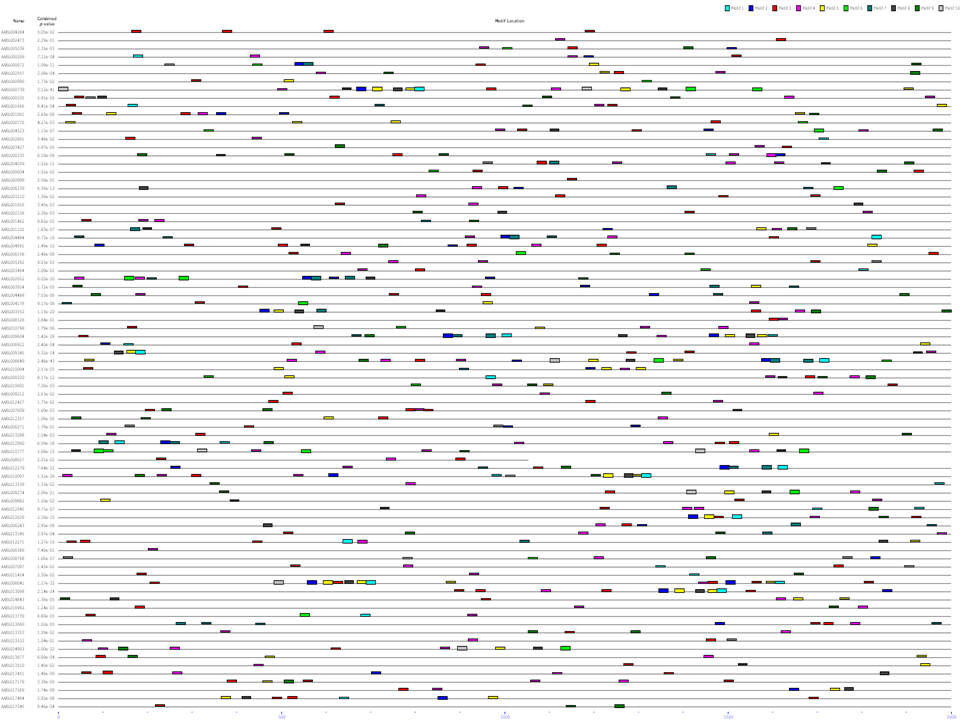

Supplement: Figure S4 — Putative CRE discovery with 83 genes in carcass samples with FPKMDENVI≥100 from 1–14 dpi. Colored boxes represent individual putative CREs and their locations in the promoters of each gene. Distances in base-pairs are provided below the schematic of each gene. (TIF) [file pone.0050512.s004.tif]

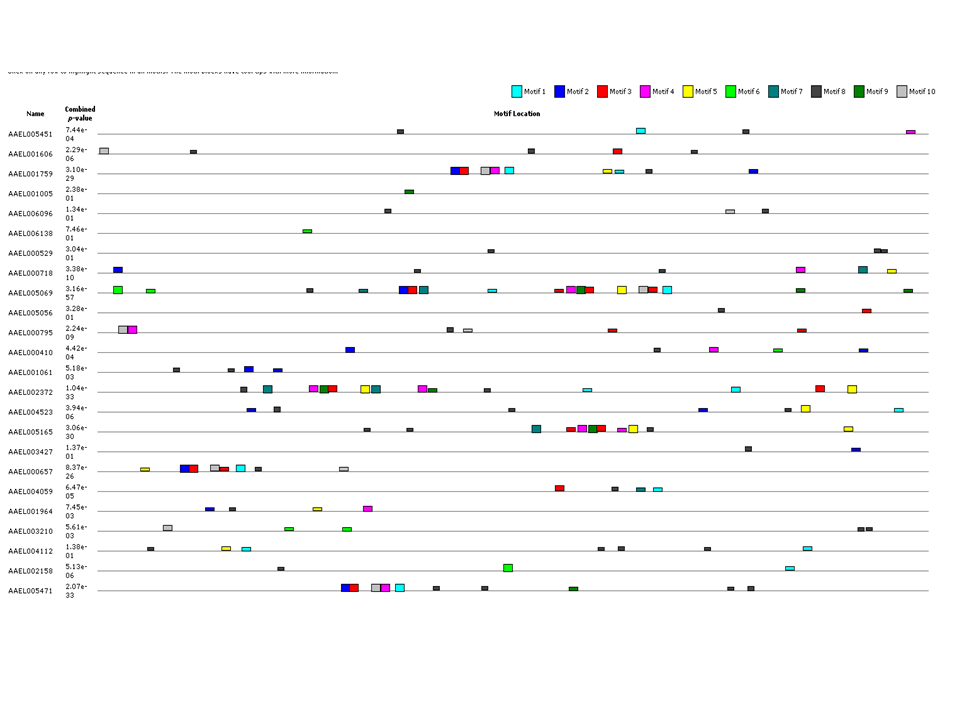

Supplement: Figure S5 — Representative putative CRE discovery with 94 genes in carcass and salivary gland samples of DENVI mosquitoes with FPKMDENVI≥100 in 14 dpi. Colored boxes represent individual putative CREs and their locations in the 2promoters of each gene. Distances in base-pairs are provided below the schematic of each gene. (TIF) [file pone.0050512.s005.tif]
